# Translation, Reliability, and Validity of the Japanese Clinical Reasoning Skills Self-Evaluation Scale: An Instrument Design Study

**DOI:** 10.7759/cureus.53177

**Published:** 2024-01-29

**Authors:** Kazuaki Naya, Hideaki Sakuramoto, Keisuke Nojima, Akira Ouchi, Saiko Okamoto, Takeya Kawasaki, Misako Kitae

**Affiliations:** 1 Nursing, Wakayama Faculty of Nursing, Tokyo Healthcare University, Wakayama, JPN; 2 Critical Care, Japanese Red Cross Kyushu International College of Nursing, Munakata, JPN; 3 Nursing, Kyoto Tachibana University, Kyoto, JPN; 4 Critical Care, Ibaraki Christian College, Hitachi, JPN; 5 Nursing, Hitachi General Hospital, Hitachi, JPN; 6 Respiratory Medicine, Ibarakihigashi National Hospital, Tokai, JPN

**Keywords:** self-evaluation scale, nursing students, nurses, japanese, clinical reasoning skills

## Abstract

Background: Currently, little evidence supports the notion that improved practical skills through simulation education are reflected in actual clinical practice and ultimately lead to positive outcomes for participants. However, by clarifying the relationship between the simulation foundation and its practicality, insights can be gained to develop educational programs to improve clinical reasoning skills. However, no clear scale is currently available in Japan.

Aims: To create a valid Japanese version of the clinical reasoning skills self-evaluation scale and evaluate its reliability and validity.

Methods: This instrument design study included 580 nursing students and nurses surveyed online from February to March 2023. The clinical reasoning skills self-evaluation scale was translated into Japanese using a back-translation method, and semantic equivalence and content validity were assessed. The content validity index was assessed using a pilot test involving 26 clinical nurses, 25 nursing students, and an expert panel. Validity and reliability were tested using a convenience sample of 580 nursing students and nurses. Reliability was assessed using internal consistency and test-retest reliability. Construct validity was assessed using confirmatory factor analysis.

Results: Cronbach's alpha for all dimensions was >0.7, and the questionnaire showed acceptable internal consistency. Test-retest reliability was evaluated using the intraclass correlation coefficient (0.674-0.797, all dimensions); the lowest value at a 95% confidence interval was 0.504 (at least moderate reliability).

Conclusion: Our scale has acceptable validity and reliability. It may help in clinical reasoning skill assessment for nurses and nursing students and aid in examining and supporting these skills.

## Introduction

Clinical reasoning, applying knowledge and skills to collect and integrate information for patient-centered diagnosis and planning, is essential for providing safe and high-quality nursing care. Dowie and Elstein defined clinical reasoning as the way clinicians think about clinical problems, including clinical judgment (determining what is wrong with the patient) and clinical decision-making (determining what should be done) [1].

Nurses are required to have clinical reasoning skills, as they provide timely patient-centered care, which is crucial for patient safety [2]. Hence, it is desirable for students and clinical nurses to utilize a conceptualized form of clinical reasoning to accurately measure their learning outcomes and confirm improvements in knowledge and skills [3,4]. However, there remains a lack of well-defined research on evidence-based and structured education and an evaluation of clinical reasoning in education programs for healthcare professions. Furthermore, the longitudinal clinical reasoning curricula adoption remains limited [5].

Currently, little evidence supports the notion that improved practical skills through simulation education are reflected in actual clinical practice and ultimately lead to positive outcomes for participants. However, by clarifying the relationship between the simulation foundation and its practicality, insights can be gained to develop educational programs to improve clinical reasoning skills.

Though research on clinical reasoning in nursing education is evolving, it lacks a clear, applicable conceptual definition in research and clinical practice [6]. Hence, it is important to understand the needs of students and nurses before graduation and design programs to improve their skills. However, no scale is currently available in Japan to assess clinical reasoning ability, which is a significant challenge.

Havola et al. developed a 26-item scale using a 5-point Likert scale based on a clinical reasoning model focused on nursing overseas [7]. This scale has been evaluated for reliability and validity and used to measure educational effectiveness. However, no clear scale is currently available in Japan. Therefore, developing a valid Japanese version of the Clinical Reasoning Skills Self-evaluation Scale is important.

This study aimed to 1) create a scientifically valid Japanese version of the Clinical Reasoning Skills Self-evaluation Scale through translation and 2) evaluate the reliability and validity of the developed scale.

## Materials and methods

Design

The original Clinical Reasoning Skills Self-evaluation Scale [7] was translated into Japanese, and its content validity, construct validity, test-retest reliability, and internal consistency were evaluated. Several cross-sectional questionnaire surveys were conducted among healthcare professionals and nursing students who were native Japanese speakers in Japan.

Translation process

Permission to translate the original instrument was obtained from Jaana-Maija Koivisto [7]. The back-translation method was followed based on previously described guidelines [8]. The translation team comprised five nursing lecturers, one nursing practitioner, and one clinical nurse. The translation process was conducted in four steps, including evaluating content validity.

STEP 1: Two nursing lecturers independently produced forward translations of the original Clinical Reasoning Skills Self-evaluation Scale from English to Japanese. Through discussions, the two translators synthesized both translations into a preliminary version. The translation team then assessed the equivalence of the original and translated versions and critically appraised whether each version was easy for healthcare professionals and nursing students to understand.

STEP 2: Two translators blinded to the original scale independently translated the forward-translated version of the Scale from Japanese to English. One translator was a native English speaker with knowledge of medical science, while the other was a native English speaker without knowledge of medical sciences. The translation team compared the two back-translated English versions with the original text and made adjustments to minimize linguistic differences. A tentative Japanese version was created after repeating this process and confirming that the linguistic differences were reduced.

STEP 3: A pilot test using the Japanese version of the Clinical Reasoning Skills Self-evaluation Scale was conducted to evaluate item clarity. A total of 51 respondents, including 26 clinical nurses and 25 nursing students, rated each item of the questionnaire on a dichotomous scale ("clear" vs. "unclear") via an online survey. The proportion of "unclear" responses for each item was calculated. If participants rated an item as "unclear" or had suggestions for improvement, they were asked to comment on those items. More than 20% of the participants rated none of the items as "unclear."

STEP 4: An expert panel, composed of five clinical nurses, three nursing lecturers, and two physicians, assessed the clarity of the scale following the same process as STEP 3. The translation team revised the items rated as "unclear" by >20% of the expert panel; each item was rated on a four-point Likert scale as follows: 1 = not relevant; 2 = unable to assess relevance; 3 = relevant but needs minor alteration; 4 = very relevant and succinct. The content validity index was determined by calculating the proportion of items with a rating of 3 or 4. A content validity index ≥0.80 was considered acceptable. The item- and scale-level content validity indices for each item and the entire scale were calculated. The translation team modified the Japanese scale until the item-level and average scale-level content validity indices were ≥0.78 and ≥0.90, respectively [8].

Construct validity, criterion validity, and test-retest reliability

Data were collected from clinical nurses and nursing students using an online survey to evaluate the construct and criterion validity of the Clinical Reasoning Skills Self-evaluation Scale between February and March 2023. Clinical nurses in Japan included those with <10 years of nursing experience in hospital settings, while nursing students included those in their first to fourth year in undergraduate nursing programs. The participants were recruited through social networking services, the community, and poster displays.

The anonymous online survey included questions about participant characteristics, such as (1) academic year, (2) years of nursing experience, (3) working unit, and (4) certifications and the Clinical Reasoning Skills Self-evaluation Scale. The mail addresses of the participants were collected to examine the intraclass correlation coefficient (ICC), and the test-retest method was used. Participants who registered their mail addresses were asked to complete the translated Clinical Reasoning Skills Self-evaluation Scale again 2 weeks after the online survey.

Sample size calculation for validity and reliability testing

Adequate statistical power is essential for detecting genuine relationships within a dataset [9]. According to the rule of thumb, a ratio of 10:1 to 20:1 for the number of samples to items in a CFA is a generally recommended ratio [9]. Consequently, we set a sample size of 520 for the confirmatory factor analysis (CFA) due to 26 items on the scale and recruited participants accordingly. For assessing reliability, particularly test-retest reliability, we used the two-way random-effects model of the ICC. Calculating the required sample size for ICC statistics using the formula by Zou [10]with a null hypothesis of 0.65, alternative hypothesis of 0.8, alpha of 0.05, and test power of 0.8, we determined that 77 patients were necessary. In addition, we estimated a 10% drop rate and recruited 90 participants.

Data analysis

Participant characteristics are expressed as numbers and percentages. Exploratory factor analysis was not performed because, to the best of our knowledge, the Clinical Reasoning Skills Self-evaluation Scale was developed using exploratory factor analysis. This study confirmed the structural equivalence between the original and Japanese scales and assessed their construct validity compared with the original measurement using CFA. Multiple fit indices, including the root-mean-square error of approximation (RMSEA), Turker-Lewis index (TLI), and comparative fit index (CFI), were used to measure the overall data model fit. An RMSEA value <0.08 was considered acceptable, while TLI and CFI >0.95 were considered acceptable [11].

The two-way random effects model of the ICC was used to assess test-retest reliability. ICCs of <0.5, 0.5 to <0.75, 0.75 to <0.9, and ≥0.9 indicated poor, moderate, good, and excellent reliability, respectively [12].

Cronbach's alpha, the most widely used internal consistency index, was used to verify the internal consistency of the questionnaire dimensions [13]. Cronbach's alpha >0.7 was considered acceptable for satisfactory internal consistency. Items with >50% missing responses in each dimension were excluded from the dataset and excluded from the analyses. All analyses were performed using the IBM Corp. Released in 2015. IBM SPSS Statistics for Windows, Version 23.0. Armonk, NY: IBM Corp., and the R-based package Jamovi v.2.3.26 (The Jamovi Project, Sydney, Australia).

Ethical considerations

The study protocol was approved by the relevant ethics committee. The survey was anonymous (no identifying data such as names, zip codes, or IP addresses were collected). All participants read the consent form and confirmed their interest in participating before starting the questionnaire. In addition, participants were informed that returning the completed questionnaires would be considered consent to participate.

## Results

Subjects

In total, 755 nurses and nursing students responded to the survey. However, 175 participants (132 nursing students and 43 nurses) provided insufficient responses and were thus excluded. Therefore, data from 580 participants, including 400 nursing students and 180 nurses, were analyzed (Table 1).

**Table 1:** Participant characteristics

Variable		
Status	Nursing student, n (%)	400 (69.0)
	Nurse, n (%)	180 (31.0)
Characteristics of the nursing students (n = 400)		
Grade in school	1, n (%)	86 (21.5)
	2, n (%)	128 (32.0)
	3, n (%)	118 (29.5)
	4, n (%)	68 (17.0)
Characteristics of the nurses (n = 180)		
Years of experience	1–3, n (%)	73 (40.5)
	4–6, n (%)	50 (27.8)
	7–10, n (%)	57 (31.7)
Section	Emergency room, n (%)	10 (5.6)
	Intensive care unit / High care unit, n (%)	51 (28.3)
	Operating room, n (%)	10 (5.6)
	General ward, n (%)	88 (48.8)
	Other, n (%)	21 (11.7)
Official position	Head nurse, n (%)	3 (1.7)
	Chief nurse / Nursing officer, n (%)	3 (1.7)
	Nursing staff, n (%)	174 (96.6)
Certification	None, n (%)	176 (97.8)
	Certified nurse specialist, n (%)	0 (0)
	Certified nurse, n (%)	4 (2.2)
	Japanese nurse practitioner, n (%)	0 (0)

Translation

The back-translated version of the Clinical Reasoning Skills Self-evaluation Scale was submitted to the original author for approval, and a pilot test was conducted with 51 participants (26 nurses and 25 nursing students). During this, three items were identified as "unclear" by >20% of the expert panel- (1) Item 11, "I can recognize a patient's need for care"; (2) Item 23, "I can support a patient's vital functions with care activities"; (3) Item 26, "I can evaluate whether a patient's clinical condition has improved, deteriorated, or is unchanged." As a result, minor revisions were made to these items based on suggestions from the expert panel while ensuring that meaning equivalence was maintained. None of the items were rated "unclear" by >20% of the participants.

Following these revisions, an expert panel reassessed the clarity of the translated scale. The content validity index for all items ranged from 0.8 to 1.0, thus confirming its validity. The translation team modified the Japanese scale until the item-level content validity index for each item was >0.78 and the average scale-level content validity index was >0.90, thus ensuring the questionnaire's content validity. The Japanese version of the Clinical Reasoning Skills Self-evaluation Scale was consequently finalized (Supplementary file S1).

Construct validity

To test construct validity, a confirmatory factor analysis using covariance structure analysis was performed. Multiple fit indices, including the RMSEA, TLI, and CFI, were used to measure the overall data-model fit. The confirmatory factor analysis indices are listed in Table 2.

**Table 2:** Confirmatory factor analysis indices Abbreviations: CFI: comparative fit index; CI: confidence interval; RMSEA: root-mean-square error of approximation; TLI: Turker-Lewis index; X2: Chi-squared.

	Indices
χ2	1079, p < 0.001
RMSEA	0.0695, 90% CI [0.0651–0.0739]
CFI	0.918
TLI	0.906

Reliability

The reliability of the scale was assessed using the test-retest method. Ninety participants (55 nursing students and 35 nurses) completed the test at both time points. Participant characteristics are listed in Table 3.

**Table 3:** Characteristics of participants assessed using the test-retest method

Variable		
Status	Nursing student, n (%)	55 (61.1)
	Nurse, n (%)	35 (38.9)
Characteristics of the nursing students (n = 55)		
Grade in school	1, n (%)	14 (25.5)
	2, n (%)	15 (27.3)
	3, n (%)	19 (34.5)
	4, n (%)	7 (12.7)
Characteristics of the nurses (n = 35)		
Years of experience	1–3, n (%)	6 (17.2)
	4–6, n (%)	11 (31.4)
	7–10, n (%)	18 (51.4)

Table 4 shows Cronbach's alpha in each dimension. Cronbach's alpha for the overall clinical reasoning skills self-evaluation scale was 0.958, and Cronbach's alphas for all dimensions were >0.7, indicating acceptable internal consistency.

**Table 4:** Cronbach's alpha in each dimension Abbreviations: CRSs: Clinical Reasoning Skills Self-evaluation Scale; CI: confidence interval

CRSs subscale	Cronbach's alpha	95% CI	F test value	p-value
Collecting information	0.833	0.691–0.867	4.927	< 0.001
Processing information	0.837	0.678–0.861	4.726	< 0.001
Identifying problems/issues	0.771	0.538–0.800	3.289	< 0.001
Establishing goals	0.867	0.504–0.786	3.068	< 0.001
Taking action	0.892	0.644–0.846	4.278	< 0.001
Evaluating outcomes	0.858	0.594–0.824	3.743	< 0.001

The ICCs for all dimensions ranged from 0.674 to 0.797, and the lowest value at the 95% confidence interval was 0.504, indicating moderate reliability (Table 5).

**Table 5:** Intraclass correlation coefficients for the test-retest method in six dimensions Abbreviations: CRSs: Clinical Reasoning Skills Self-evaluation Scale; CI: confidence interval; ICC: intraclass correlation coefficient

Total and subscale CRSs scores	ICC	95% CI	F test value	p-value
Total score	0.828	0.739–0.887	5.820	< 0.001
Collecting information	0.797	0.691–0.867	4.927	< 0.001
Processing information	0.788	0.678–0.861	4.726	< 0.001
Identifying problems/issues	0.696	0.538–0.800	3.289	< 0.001
Establishing goals	0.674	0.504–0.786	3.068	< 0.001
Taking action	0.766	0.644–0.846	4.278	< 0.001
Evaluating outcomes	0.733	0.594–0.824	3.743	< 0.001

## Discussion

This study developed a Japanese version of the Clinical Reasoning Skills Self-evaluation Scale using a back-translation protocol, expert clarity and validity evaluations, and pilot tests. The findings confirmed the reliability and validity of the Japanese-translated scale. The translation followed standard methods, and face validity, content validity, and understandability were established. Back-translation, performed by two translators with different backgrounds, considering the differences in medical terminology and subtle nuances, was used to correct content variations. Finally, the face validity, relevant validity, and understandability were evaluated and established using a pilot test and a multidisciplinary expert panel.

The construct validity was considered acceptable. Previous studies suggested that an RMSEA value <0.05 indicates a "close fit" and that a value <0.08 suggests a reasonable model-data fit [14]. In addition, [15] recommended that a TLI >0.90 indicates an acceptable fit. However, Hu and Bentler suggested that an RMSEA <0.06 and CFI and TLI >0.95 generally indicate a relatively good model-data fit [11]. Thus, applying the RMSEA, CFI, and TLI are heavily contingent on the set of cutoff criteria. In this study, the RMSEA was 0.0695, and the CFI and TLI were >0.90, indicating that the construct validities were within acceptable limits.

Furthermore, the ICCs of each factor of the scale created in this study were slightly lower for "Identifying problems/issues" and "Establishing goals" than for other factors. However, all factors were interpreted as substantial (0.675-0.797), indicating acceptable inter-rater reliability. As an index of test-retest reliability, the ICC was at least moderate. However, the total score was >0.8, indicating good reliability. As no previous studies have evaluated the ICC for the Clinical Reasoning Skills Self-evaluation Scale, the criteria of [12] were used. ICC values <0.5, 0.5 to <0.75, 0.75 to <0.9, and ≥ 0.9 indicated poor, moderate, good, and excellent reliability, respectively.

In addition, the internal consistency of the Clinical Reasoning Skills Self-evaluation Scale was acceptable because the lowest Cronbach's alpha was 0.771 (0.771-0.892), which is considered acceptable [13].

Strengths and limitations of the work

This scale is easy for nursing students and nurses to respond to because it contains only 26 items, and the questions are simple. Therefore, it can be used to evaluate both basic and current nursing education programs. It can also be used in practice and research for a seamless transition from basic to current nursing education.

This study has some limitations. First, the participants included both nursing students and nurses; however, nursing students constituted most participants. This was also the case for the test-retest method. Therefore, the results are primarily based on nursing students' perspectives, and caution should be exercised when generalizing the results. In addition, the test-retest method involved fewer than six study participants with <3 years of nursing experience. This bias may have had a small impact on the reliability assessment. Second, regarding the reliability of the measurement scale, high Cronbach's alpha values were obtained for all dimensions (0.771-0.892). However, Cronbach's alpha values for the dimensions identifying problems/issues and establishing goals were lower than those for the other dimensions, raising reliability concerns. In addition, the reliability ratings based on the test-retest method showed constant ICC values for all dimensions but with moderate reliability (0.674-0.797). These results suggest that certain dimensions of the measurement scale may have reliability challenges. Third, criterion validity was not assessed due to the absence of a Japanese version of the scale to serve as a reference. Studies evaluating criterion validity, utilizing tools such as the Nurse Clinical Reasoning Competence scale [16], are necessary to determine the scale's ability to predict actual clinical reasoning performance.

Recommendations for further research

The clinical reasoning skills of nurses in Japan are crucial. Therefore, considerable education is provided to both nurses and nursing students. However, to date, no clear measure of the clinical reasoning abilities of nurses in Japan exists. The novelty of this study lies in verifying the reliability and validity of the Japanese Clinical Reasoning Skills Self-evaluation Scale.

Using this scale, it will be possible to examine and establish support for nurses and nursing students in developing their clinical reasoning skills.

Furthermore, this study can improve the quality of nursing education in Japan. Havola et al. found that the original scale version clearly improved clinical reasoning skills in simulation games when nursing students engaged in virtual reality simulations [7]. Since the spread of the coronavirus disease (2019), educational methods incorporating information and communication technology have been implemented in Japan. Therefore, examining how these factors affect nursing students' clinical reasoning skills is important. Additionally, measuring the clinical reasoning skills of Japanese nurses and nursing students can make international comparisons possible. Future research is necessary to continue validating this study with more nurses as participants.

## Conclusions

The findings of this study suggest that the Japanese version of the Clinical Reasoning Skills Self-evaluation Scale has acceptable validity and reliability. This scale may help assess clinical reasoning skills in nurses and nursing students and aid in the examination and establishment of support for nurses and nursing students to develop clinical reasoning skills.

## Appendices

Supplementary file S1

**Table 6:** Japanese version of the Clinical Reasoning Skills Self-evaluation Scale

質問項目	非常にそう思う	そう思う	どちらでもない	そう思わない	全くそう思わない
情報収集					
患者の記録やその他の文書から情報を収集することができる	5	4	3	2	1
問診により患者の情報を収集することができる	5	4	3	2	1
観察により患者の情報を収集することができる	5	4	3	2	1
測定により患者の情報を収集することができる（例：血圧、心拍数、呼吸数、血糖値など）	5	4	3	2	1
患者の状態を評価するために、ABCDEアプローチなどの理論的枠組みを適用することができる	5	4	3	2	1
情報の処理					
収集した情報を解釈することができる	5	4	3	2	1
さらなる情報の必要性に気づくことができる	5	4	3	2	1
必須な情報と必須でない情報を区別することができる	5	4	3	2	1
自分の判断が患者の状態に及ぼす影響を予測することができる	5	4	3	2	1
患者の状態に関して原因と結果の関係をアセスメントすることができる	5	4	3	2	1
問題・課題の抽出					
患者へのケアの必要性を認識することができる	5	4	3	2	1
看護診断を行うことができる（＊看護診断を使用していない場合は看護問題の抽出）	5	4	3	2	1
患者の状態の変化に気づくことができる	5	4	3	2	1
患者のニーズに優先順位をつけることができる	5	4	3	2	1
目標の設定					
看護の方針を設定することができる	5	4	3	2	1
必要なケアを計画することができる	5	4	3	2	1
患者へのケアを決定することができる	5	4	3	2	1
必要な際には、患者へのケアを迅速に決定することができる	5	4	3	2	1
実践					
症状に対するケアを実施することができる	5	4	3	2	1
複数のケアの選択肢の中からケアを選ぶことができる	5	4	3	2	1
患者の状態に合わせたケアを選択することができる	5	4	3	2	1
状態が悪化した患者のケアを実施することができる	5	4	3	2	1
患者の生命を維持するためのケアを実施することができる	5	4	3	2	1
アウトカムの評価					
ケアが計画通りに実施できたか評価することができる	5	4	3	2	1
患者のニーズに対するケアの成果を評価することができる	5	4	3	2	1
患者の状態変化（改善・悪化・変化なし）を評価することができる	5	4	3	2	1
